# Influence of changes in serum uric acid levels on renal function in elderly patients with hypertension: a retrospective cohort study with 3.5-year follow-up

**DOI:** 10.1186/s12877-016-0209-2

**Published:** 2016-02-03

**Authors:** Fan Lin, Hailin Zhang, Feng Huang, Hui Chen, Chunjin Lin, Pengli Zhu

**Affiliations:** Department of Geriatric Medicine, Fujian Provincial Hospital, Fujian Institute of Clinical Geriatrics, Fuzhou, 350001 China; Department of Cardiology, Fujian Provincial Hospital, Fuzhou, 350001 China; Fujian Medical University, Fuzhou, 350001 China

**Keywords:** Essential hypertension, Serum uric acid, Renal function

## Abstract

**Background:**

Hyperuricemia is closely related to renal diseases. Therefore, the aim of this study was to explore the relationship between the longitudinal changes in serum uric acid and the estimated glomerular filtration rate (eGFR) in a cohort of elderly hypertensive patients.

**Methods:**

Eighty hundred and thirty-seven re-hospitalized patients with hypertension were included in this retrospective cohort study. Multiple regression analysis was used to investigate the relationship between changes in serum uric acid and renal function after 3.5 years follow-up.

**Results:**

The average age at baseline was 69.0+/-10.0 years, and the average follow-up duration was 3.5 years. Multiple linear regression analysis showed that the baseline uric acid levels had a linearly negative correlation with baseline eGFR (*P* < 0.01), after adjustment for age, gender, blood pressure, and body mass index, et al. An increase of 100 μmol/L baseline uric acid level resulted in a decrease of 5.684 ml/min/1.73 m^2^ in eGFR [95 % confidence interval (CI): 7.735-3.633]. Patients with increased uric acid levels had higher risk of renal function decline over the follow-up period, with an adjusted odds ratio of 1.639 (95 % CI: 1.129-2.378, *P* = 0.009) , whereas eGFR was remained unchanged in patients with hyperuricemia at baseline and with normal uric acid level 3.5-year later.

**Conclusions:**

Longitudinal changes in uric acid levels were independently associated with the renal function decline in elderly patients with hypertension. Uric acid level should be considered in hypertension management in the elderly.

## Background

Uric acid is an endogenous end product of metabolism of purine nucleotide. Due to lack of uricase encoding genes, humans are unable to convert uric acid to soluble allantoin, leading to higher levels of serum uric acid in humans than in most of the other mammals that have uricase encoding genes [1]. Hyperuricemia shows similar epidemiological trends as hypertension, chronic renal disease, and type 2 diabetes [2–5], and it is a risk factor for all above diseases. Hyperuricemia is closely related to renal function decline both in healthy subjects [6–8] and in patients with other related diseases [9]. The risk of albuminuria significantly increased in patients with hyperuricemia by 1.27 times in healthy Korean men (mean age, 52.8 years) with a 5-year follow-up [6]. In a prospective study among a community-based Chinese population (mean age, 59.1 years) with the estimated glomerular filtration rate (eGFR) ≥60 ml/min per 1.73 m^2^, baseline uric acid levels were independently associated with an increased risk of renal function decline, even they still in the ‘normal’ range [7]. Another prospective study among an elderly Chinese population (mean age, 74.5 years) in Taiwan had the similar results, with the mean follow-up period of 32.4 months [8]. Hyperuricemia predicted a significantly 1.43 times higher risk for kidney disease among US veterans with gouty arthritis [9].

Although the relationship between serum uric acid and renal function decline has attracted increasing attention, little is known about the importance of uric acid level with regard to renal damage in elderly patients with hypertension. Ohta Y et al had reported that an increased uric acid level promoted the renal function decline in patients with hypertension over a 10-year observation period [10]. Our earlier study found that hyperuricemia was an independent risk factor for poor control of blood pressure during hospitalization in elderly patients with hypertension [11], and subjects in our study were much older and had more comorbidity compared with that in the study of Ohta Y et al. Therefore, the aim of this study was to determine whether longitudinal changes in uric acid level were associated with the renal function in this cohort of elderly hypertensive patients. To the best of our knowledge, this was the first study focused on whether a decrease in serum uric acid might slow down the progression of renal damage in elderly patients with hypertension, independent of age, diabetes, and chronic kidney disease. The result may play an important clinical role in the preservation of renal function for elderly patients with hypertension.

## Methods

### Participants

In our earlier study, a total of 1648 inpatients with hypertension were enrolled [11], among whom 865 patients were re-admitted to Fujian Provincial Hospital between August 2010 and July 2014. The study was in compliance with the ethics regulations of the Helsinki Declaration, and the protocol was approved by the institutional review board of Fujian Provincial Hospital. All patients gave oral informed consent to take part in the original study and for their data to be used. The inclusion criterion was the diagnosis of essential hypertension on hospital admission. Patients were excluded for any of the following reasons: hypertension emergencies requiring intravenous infusion of antihypertensive drugs, severe cardiac insufficiency (grade IV according to the New York Heart Association), acute coronary syndrome, acute cerebrovascular accident, unconsciousness due to any reason, infectious disease, hemodynamic instability due to any reason, receiving diuretics or allopurinol within 2 weeks prior to admission, incomplete data on blood pressure or serum uric acid. Overall, 28 patients were excluded and 837 patients were included in the follow-up study with an average follow-up duration of 3.5 years.

### Collection of clinical data

The medical history and clinical data were collected at baseline and readmission, respectively, including medical history, family history, number and dosage of medications (antihypertensive agents, antilipemic agents, antidiabetic agents, etc.). Blood pressure in the right arm was measured twice in the supine position, using a manual sphygmomanometer after at least 10 min of rest, and the average of the two readings was used for analysis. Any discomfort was avoided during the blood pressure measurements. The fasting forearm vein blood (5 ml) was collected in the morning, and an automatic clinical chemistry analyzer (Dxc800; Beckman Coulter, CA, 92821) was used to detect the levels of blood glucose, uric acid, creatinine, triacylglycerol, total cholesterol, high-density ipoprotein cholesterol (HDL-C), and low-density lipoprotein-cholesterol (LDL-C).

### Diagnostic definition

Hypertension was defined as systolic blood pressure (SBP) ≥140 mmHg and/or diastolic blood pressure (DBP) ≥90 mmHg without the use of anti-hypertensive drugs, or currently taking antihypertensive drugs. Hyperuricemia was defined as the level of uric acid >420 μmol/L in men and >360 μmol/L in women with normal purine diet. Diabetes was defined as diabetic symptoms with the level of random blood glucose ≥11.1 mmol/L, or fasting blood glucose ≥7.0 mmol/L, or blood glucose ≥11.1 mmol/L at 2 h after loading glucose, or previously diagnosed diabetes. Patients without diabetic symptoms required a review on another day. eGFR was calculated using Cockcroft-Gault formula recommended by the National Kidney Foundation: eGFR in men = [(140-age) × weight (kg)]/[0.818 × serum creatinine (μmol/L)], eGFR in women = [(140-age) × weight (kg)]/[0.818 × serum creatinine (μmol/L) × 0.85]. Antihypertensive therapeutic intensity score was defined as the daily dose of antihypertensive agents divided by the maximum FDA approved dose, summed across all medication classes [12]. Body mass index (BMI) was calculated as weight (kg) / height squared (m^2^).

### Statistical analysis

All the data were analyzed using the Statistical Package for the Social Sciences, version 19.0 (SPSS, Inc., Chicago, IL, USA). All continuous variables were approximately normal distributed, and were expressed as mean ± standard deviation. Categorical data were expressed as counts and percentages with the Chi-square test used to compare differences (e.g. gender, diabetes, administration of statins, pressure-target rate) between groups. Paired sample *t*-test was used to compare differences between uric acid reduced group and uric acid increased group both at baseline and 3 years later. Patients were divided into four groups according to quartiles of baseline uric acid levels, and one-way analysis of variance (ANOVA) was used to estimate the association of uric acid levels with other cardiovascular risk factors (e.g. age, hypertension duration, BMI, SBP, DBP, fasting blood glucose, triacylglycerol, total cholesterol, HDL-C, LDL-C, and eGFR) and linear trend test was conducted afterwards. A multiple linear regression model was constructed to study the independent association between eGFR and serum uric acid, after adjustment for other risk factors such as gender, hypertension duration, BMI, blood pressure, antihypertensive therapeutic intensity score, fasting blood glucose, and lipid profiles. The value of Durbin-Watson was approximated to 2 and the standardized residuals of eGFR were normally distributed. A binary logistic regression model was used to determine the relationship of longitudinal changes in uric acid and the renal function decline, after controlling for other potential confounders. The Hosmer and Lemeshow goodness-of-fit test was used for logistic regression (*P* > 0.05). A two-sided *P* value < 0.05 was considered statistically significant.

## Results

**Fig. 1:**
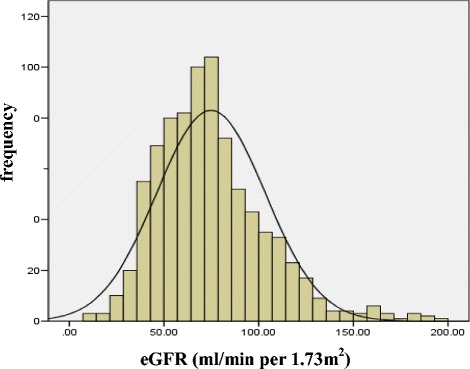
eGFR distribution of the study subjects at baseline. Note: eGFR: estimated glomerular filtration rate

**Fig. 2:**
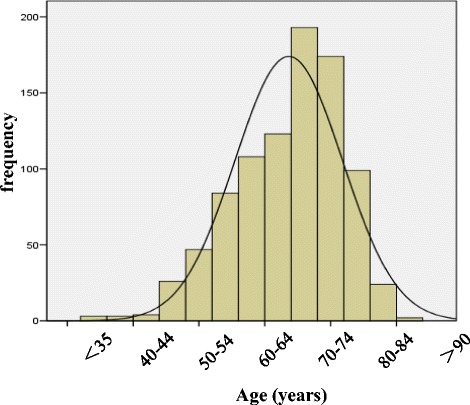
Age distribution of the study subjects by 5-year group at baseline

The distribution of eGFR and age of all subjects at baseline were showed in Fig. 1 and Fig. 2, respectively. The average eGFR and average age was 74.78 ± 28.74 ml/min per 1.73 m^2^ and 69.0 ± 10.0 years, respectively. The average follow-up duration was 3.5 years, and the average of uric acid level was 349.95 ± 102.06 μmol/L at baseline and 351.16 ± 114.23 μmol/L at 3.5 years later. There was no significant difference in uric acid level between baseline and follow-up among all patients (*P* = 0.758).

### Comparison between patients with hyperuricemia and patients with normal serum uric acid at baseline

Baseline clinical characteristics of the two groups were presented in Table 1. Baseline BMI, antihypertensive therapeutic intensity score, triacylglycerol and LDL-C were higher in hyperuricemia group (all *P <* 0.05), whereas baseline eGFR was lower (*P <* 0.01). No significant differences were observed between the two groups regarding the age, gender, diabetes history, blood pressure, fasting blood glucose or total cholesterol.

**Table 1:** Clinical characteristics between subjects with hyperuricemia and with normal uric acid at baseline

	Normal uric acid (n = 609)	Hyperuricemia (n = 256)	*P*
Age (years)	68.96 ± 10.01	68.95 ± 10.44	0.996
Men (n, %)	313 (49.87 %)	142 (47.41 %)	0.294
Hypertension duration (years)	10.8 ± 8.1	9.0 ± 7.3	0.301
Diabetes(n, %)	186 (25.06 %)	77 (25.93 %)	0.892
chronic kidney disease (n, %)	151 (38.23 %)	240 (24.90 %)	<0.001
Body mass index (kg/m^2^)	24.43 ± 3.50	25.43 ± 3.66	<0.001
Antihypertensive therapeutic intensity score	0.89 ± 0.79	1.06 ± 0.88	0.010
Systolic blood pressure (mmHg)	145.79 ± 18.52	146.79 ± 20.58	0.484
Diastolic blood pressure (mmHg)	79.52 ± 12.57	80.75 ± 13.09	0.198
Fasting blood glucose (mmol/L)	5.84 ± 1.73	5.63 ± 1.42	0.066
Triacylglycerol (mmol/L)	1.58 ± 1.17	1.92 ± 1.18	<0.001
Total cholesterol (mmol/L)	4.80 ± 1.92	5.06 ± 1.30	0.061
HDL-C (mmol/L)	1.22 ± 0.41	1.17 ± 0.35	0.092
LDL-C (mmol/L)	2.85 ± 0.94	3.09 ± 1.16	0.007
Uric acid (umol/L)	298.66 ± 67.17	466.23 ± 66.61	<0.001
eGFR (ml/min/1.73 m^2^)	76.91 ± 28.76	68.95 ± 10.44	0.001

*Note:* 1 mmHg = 0.333 kPa; HDL-C: high-density lipoprotein-cholesterol; LDL-C: low-density lipoprotein-cholesterol; eGFR: estimated glomerular filtration rate

### Association between serum uric acid and eGFR at baseline

Patients then were divided by quartiles based on the baseline uric acid level, and the clinical data were shown in Table 2. The hypertension duration, BMI, antihypertensive therapeutic intensity score, and triacylglycerol level were increased in accordance with the increase of uric acid level, while HDL-C level and eGFR were decreased. There was a linear decrease in eGFR with the increases of uric acid level, after adjustment for age, gender, hypertension duration, BMI, SBP, antihypertensive therapeutic intensity score, triacylglycerol, HDL-C, and LDL-C. Multiple regression analysis showed that an increase in the uric acid level by 100 μmol/L led to a decrease of 5.684 ml/min per 1.73 m^2^ in eGFR [95 % confidence interval (CI): 7.735-3.633, *P <* 0.01] (Table 3).

**Table 2:** Characteristics of study population in the stratified quartiles based on the baseline level of serum uric acid

	Q_1_ (n = 233)	Q_2_ (n = 232)	Q_3_ (n = 200)	Q_4_ (n = 200)	*P*
Age (years)	69.5 ± 10.1	69.2 ± 9.9	67.4 ± 9.7^*^	69.6 ± 10.7	0.102
Men (n, %)	79 (33.0)	107 (46.1)^**^	127 (63.5)^**^	142 (71.0)^**^	<0.001
Hypertension duration (years)	8.6 ± 8.2	9.0 ± 8.8	10.1 ± 8.3^*^	11.4 ± 9.8^**^	<0.001
Diabetes (n, %)	75 (32.2)	69 (29.7)	59 (29.5)	60 (30.0)	0.921
Body mass index (kg/m^2^)	24.3 ± 3.7	24.2 ± 3.4	25.1 ± 3.3^*^	25.5 ± 3.8^**^	<0.001
Administration of statins (n, %)	179 (76.8)	183 (78.9)	168 (84.0)	155 (77.5)	0.266
Antihypertensive therapeutic intensity score	0.9 ± 0.8	0.9 ± 0.8	0.9 ± 0.8	1.2 ± 0.9^**^	<0.001
Systolic blood pressure (mmHg)	145.9 ± 18.4	146.2 ± 18.7	144.9 ± 19.7	147.3 ± 20.1	0.673
Diastolic blood pressure (mmHg)	79.1 ± 12.8	79.6 ± 12.6	80.3 ± 12.7	80.7 ± 12.8	0.582
Fasting blood glucose (mmol/L)	6.04 ± 1.98	5.72 ± 1.62	5.74 ± 1.46	5.58 ± 1.38^*^	0.027
Triacylglycerol (mmol/L)	1.48 ± 1.18	1.62 ± 1.25	1.75 ± 1.16^*^	1.90 ± 1.07^**^	0.004
Total cholesterol (mmol/L)	4.95 ± 2.77	4.77 ± 1.21	4.87 ± 1.13	4.92 ± 1.26	0.761
HDL-C (mmol/L)	1.28 ± 0.37	1.23 ± 0.48	1.16 ± 0.33^**^	1.15 ± 0.35^**^	0.002
LDL-C (mmol/L)	2.86 ± 0.95	2.88 ± 0.99	2.94 ± 0.96	3.01 ± 1.14	0.491
Uric acid (umol/L)	230.73 ± 43.48	315.12 ± 20.06^**^	383.37 ± 21.13^**^	488.50 ± 57.64^**^	<0.001
eGFR (ml/min per 1.73 m^2^)	78.46 ± 27.98	75.64 ± 27.39	75.02 ± 31.68	68.91 ± 27.70^**^	0.008

*Note:* *HDL-C* high-density lipoprotein-cholesterol, *LDL-C* low-density lipoprotein-cholesterol, *eGFR* estimated glomerular filtration rate. Compared with Q_1_ group ^*^ *P* < 0.05, ^**^ *P* < 0.01

**Table 3:** Effect of level of serum uric acid on eGFR using linear regression

	*β* (SE)	95 % CI	*t*	*P*
Model 1	-3.368 (0.989)	-5.309, -1.427	-3.406	0.014
Model 2	-4.274 (0.984)	-6.205, -2.344	-4.345	<0.001
Model 3	-5.684 (1.044)	-7.735, -3.633	-5.442	<0.001

*Note:* *eGFR* estimated glomerular filtration rate, *SE* standard error, *CI* confidence interval. Model 1 was not adjusted for any factor. Model 2 was adjusted for age and gender. Model 3 was adjusted for age, gender, body mass index, systolic blood pressure, antihypertensive therapeutic intensity score, triacylglycerol, high-density lipoprotein–cholesterol, and low-density lipoprotein-cholesterol. The adjusted *R* ^2^ for the multiple linear regression model was 0.416

### Association of longitudinal changes in serum uric acid with eGFR decline 3.5 years later

According to the changes of uric acid throughout the follow-up period, patients were divided into uric acid reduced group (n = 399) and uric acid increased group (n = 391). There were 47 patients with the uric acid level unchanged. As shown in Table 4, The proportion of patients with diabetes significantly increased (*P <* 0.01), and the antihypertensive therapeutic intensity score significantly decreased (*P <* 0.05) 3.5 years later in both groups. Besides, eGFR in uric acid increased group was significantly reduced (*P <* 0.01) 3.5 years later compare with that in uric acid reduced group, whereas baseline eGFR was similar between two groups. From the data, we also found that the patients in uric acid increased group had longer hypertension duration (*P <* 0.01). The binary logistic regression showed that the odds ratio of eGFR reduction in uric acid increased group was 1.639 (95 % CI: 1.129-2.378, *P* = 0.009).

**Table 4:** Characteristics of study population between uric acid reducing group and uric acid increasing group

	Uric acid reduced group (n = 399)			Uric acid increased group (n = 391)		
	Baseline	At 3 years	*P*	Baseline	At 3 years	*P*
Age (years)	68.2 ± 10.5	71.7 ± 10.5	<0.001	70.0 ± 9.5	73.7 ± 9.6	<0.001
Men (n, %)	211 (52.9)	-	-	206 (52.7)	-	-
Hypertension duration (years)	9.2 ± 8.4	-	-	11.4 ± 9.6	-	-
Diabetes (n, %)	110 (27.6)	131 (32.8)	0.001	131 (33.5)	153 (39.1)	<0.001
Body mass index (kg/m^2^)	24.59 ± 3.34	24.26 ± 3.61	0.009	25.11 ± 3.87	25.18 ± 4.04	0.558
Administration of statins (n, %)	321 (80.5)	384 (96.2)	<0.001	336 (85.9)	353 (90.3)	0.006
Antihypertensive therapeutic intensity score	0.94 ± 0.84	0.81 ± 0.62	0.003	0.95 ± 0.85	0.84 ± 0.63	0.020
Systolic blood pressure (mmHg)	145.43 ± 18.56	141.50 ± 19.36	0.003	131.96 ± 14.88	135.66 ± 17.20	0.001
Diastolic blood pressure (mmHg)	80.32 ± 13.30	77.49 ± 12.14	<0.001	73.83 ± 7.89	72.73 ± 11.46	0.101
Pressure-target rate (n, %)	122 (30.6)	167 (41.9)	<0.001	257 (65.7)	139 (35.6)	0.001
Fasting blood glucose (mmol/L)	5.70 ± 1.64	6.11 ± 1.92	<0.001	5.87 ± 1.73	6.05 ± 1.72	0.044
Triacylglycerol (mmol/L)	1.72 ± 1.07	1.48 ± 0.93	<0.001	1.65 ± 1.28	1.63 ± 1.05	0.792
Total cholesterol (mmol/L)	4.94 ± 1.15	4.58 ± 1.28	<0.001	4.81 ± 2.31	4.64 ± 1.22	0.143
HDL-C (mmol/L)	1.22 ± 0.35	1.20 ± 0.42	0.264	1.20 ± 0.44	1.17 ± 0.37	0.186
LDL-C (mmol/L)	3.02 ± 1.08	2.85 ± 1.06	0.010	2.80 ± 0.93	2.89 ± 1.00	0.094
Uric acid (μmol/L)	380.88 ± 100.04	301.19 ± 86.95	<0.001	318.62 ± 94.68	402.20 ± 116.64	<0.001
eGFR (ml/min per 1.73 m^2^)	73.36 ± 29.61	72.33 ± 27.01	0.539	74.51 ± 26.87	66.24 ± 28.25	<0.001

*Note:* *eGFR* estimated glomerular filtration rate, *HDL-C* high-density lipoprotein cholesterol, *LDL-C* low-density lipoprotein-cholesterol. Blood pressure was calculated as the average of blood pressure on admission and at discharge

Furthermore, patients were divided into four groups for further analysis. Four hundred and sixty-five patients had normal uric acid levels throughout the follow-up period (292.53 ± 67.57 μmol/L and 290.22 ± 65.82 μmol/L, respectively, defined as N-N group); 116 patients had normal serum uric acid levels at baseline and had hyperuricemia later (330.09 ± 59.17 μmol/L and 460.91 ± 82.76 μmol/L, respectively, defined as N-H group); 114 patients had hyperuricemia at baseline and normal uric acid level later (461.25 ± 67.08 μmol/L and 317.11 ± 72.99 μmol/L, respectively, defined as H-N group); 142 patients had hyperuricemia throughout the follow-up period (471.91 ± 65.95 μmol/L and 493.18 ± 100.33 μmol/L, respectively, defined as H-H group). The eGFR significantly decreased in the N-H group (*P* = 0.002), H-H group (*P* = 0.021), and N-N group (*P* = 0.035), while the eGFR remained unchanged in the H-N group (*P* = 0.705) (Table 5).

**Table 5:** Comparison of eGFR between baseline and follow-up categorized by longitudinal changes in uric acid level

	Baseline (ml/min per 1.73 m^2^)	At 3.5 years (ml/min per 1.73 m^2^)	*P*
N-N	76.13 ± 27.75	72.64 ± 27.06	0.035
N-H	75.59 ± 28.27	66.32 ± 27.12	0.002
H-N	69.90 ± 30.45	68.78 ± 24.89	0.705
H-H	68.10 ± 27.34	61.94 ± 30.58	0.021

*Note:* *eGFR* estimated glomerular filtration rate, *N-N group* patients with normal uric acid throughout the follow-up period, *N-H group* patients with normal uric acid at baseline and hyperuricemia 3.5 years later, *H-N group* patients with hyperuricemia at baseline and normal uric acid 3.5 years later, *H-H group* patients with hyperuricemias throughout the follow-up period

## Discussion

Our study reported for the first time that a decrease in serum uric acid might slow down the progression of renal damage in elderly patients with hypertension. In this study, all subjects were elderly hypertensive patients (aged about 70 years) with other comorbidity such as overweight, diabetes and dyslipidemia. Patients with hyperuricemia usually had lower eGFR, along with higher hypertension duration, BMI, antihypertensive therapeutic intensity score and triacylglycerol. So we performed the multiple linear regressions to determine the independent association between uric acid level and eGFR. The result showed that serum uric acid was negatively linearly correlated to eGFR independent of sex, BMI, antihypertensive drugs, and lipid profiles. An increase of 100 μmol/L baseline uric acid level led to a decrease of 5.684 ml/min per 1.73 m^2^ in eGFR.

In the analysis of longitudinal data we found that patients with increased uric acid level had a significantly greater eGFR decline than those patients with decreased uric acid level, although there was no significant difference in eGFR between two groups at baseline. The risk of deterioration in renal function significantly increased 1.639 times in patients with increased serum uric acid. On the contrary, patients with hyperuricemia at baseline but normal uric acid 3.5 years later (H-N Group) maintained stable eGFR, indicating that a decrease in serum uric acid level would help to slow down deterioration of renal function.

There is a reciprocal causal relationship between hyperuricemia and renal disease. On one hand, reduction of uric acid through renal excretion causes hyperuricemia. On the other hand, hyperuricemia induces renal injury via a crystal-independent mechanism involving renal vasoconstriction mediated by endothelial dysfunction and activation of renin-angiotensin system [13]. Recent studies have shown that elevated serum uric acid is an independent risk factor for renal impairment irrespective of animal models, healthy population, or patients with hypertension, diabetes, or chronic renal diseases [14–17]. Shan Y et al. reported that hypertension, diabetes and hyperuricaemia were three independent risk factors for chronic kidney disease in adults over 40 years in Central China [18]. However, prospective studies on the effect of hypouricemic therapy on renal function are rare. Malaguarnera et al. conducted a randomized self-cross controlled study in elderly patients with hyperuricemia who received urate oxidase or placebo. The patients treated with urate oxidase had a significant reduction in serum uric acid and creatinine, and had an increase in creatinine clearance [19]. Goicoechea et al. found that allopurinol treatment slowed down the deterioration of renal function in patients with chronic kidney disease [20]. Another study also showed the benefits of allopurinol therapy on eGFR and blood pressure hyperuricemic patients with normal renal function [21]. A retrospective, observational cohort study showed that hyperuricemia was not an independent factor for renal progression in autosomal dominant polycystic kidney disease. However, the correction of hyperuricemia may attenuate renal function decline in patients with mild renal insufficiency [22].

As mentioned above, subjects in this study were elderly hypertensive patients with other comorbidity. As shown in Table 4, the proportion of patients with diabetes increased in both groups, indicating disorders of glucose metabolism with aging. Besides, antihypertensive therapeutic intensity score was decreased in all subjects, showing the lack of compliance with hypertension treatment in the elderly. Both dyslipidemia and hyperuricemia are common in the elderly, which are the manifestation of metabolism disorders. Obesity has been demonstrated as a potentially reversible risk factor for the development of GFR decline, which may be mediated by the presence of cardiovascular risk factors including diabetes, hypertension and dyslipidemia [23]. Our result showed a decrease in the levels of lipid profiles (including triacylglycerol and total cholesterol), reflecting the popularity in the use of statins. Age itself is an independent risk factor for the decline in renal function. Our study demonstrated that reduction of uric acid level could preserve renal function independent of age, diabetes, dyslipidemia and administration of statins. The result indicates that more attention should be paid on the serum uric acid for better hypertension management, especially in reducing the incidence of chronic kidney disease in the elderly.

As a retrospective cohort study, this study had inevitable limitations. First, this study could not explain the causal relationship between hyperuricemia and renal function. Second, only that information during hospitalization was collected and 3.5-year was a relatively short follow-up period. Moreover, diuretics and allopurinol agents are widely used. Though patients taking these two types of drugs recently were excluded, the medication history between two hospitalizations might still have an impact on uric acid level and renal function. Therefore, the conclusions of this study should not be extended to all patients with hypertension. More prospective cohort studies are required to clarify the protective function of reduction of uric acid level on renal function in patients with hypertension.

## Conclusions

Increased serum uric acid levels contributed to the eGFR decline in elderly hypertensive patients. Reduction of serum uric acid may preserve renal function independent of age, diabetes and dyslipidemia. Hypouricemic therapy should be considered in hypertension management in elderly patients with hyperuricemia.

## References

[1] Oda M, Satta Y, Takenaka O, Takahata N (2002). Loss of urate oxidase activity in hominoids and its evolutionary implications. Mol Biol Evol.

[2] Kodama S, Saito K, Yachi Y, Asumi M, Sugawara A, Totsuka K (2009). Association between serum uric acid and development of type 2 diabetes. Diabetes Care.

[3] Viazzi F, Leoncini G, Vercelli M, Deferrari G, Pontremoli R (2011). Serum uric acid levels predict new-onset type 2 diabetes in hospitalized patients with primary hypertension: the MAGIC study. Diabetes Care.

[4] Choi HK, Atkinson K, Karlson EW, Curhan G (2005). Obesity, weight change, hypertension, diuretic use, and risk of gout in men: the health professionals follow-up study. Arch Intern Med.

[5] Johnson RJ, Kang DH, Feig D, Kivlighn S, Kanellis J, Watanabe S (2003). Is there a pathogenetic role for uric acid in hypertension and cardiovascular and renal disease. Hypertension.

[6] Oh CM, Park SK, Ryoo JH (2014). Serum uric acid level is associated with the development of microalbuminuria in Korean men. Eur J Clin Invest.

[7] Zhang L, Wang F, Wang X, Liu L, Wang H (2012). The association between plasma uric acid and renal function decline in a Chinese population-based cohort. Nephrol Dial Transplant.

[8] Yen CJ, Chiang CK, Ho LC, Hsu SH, Hung KY, Wu KD (2009). Hyperuricemia associated with rapid renal function decline in elderly Taiwanese subjects. J Formos Med Assoc.

[9] Krishnan E, Akhras KS, Sharma H, Marynchenko M, Wu E, Tawk RH (2013). Serum urate and incidence of kidney disease among veterans with gout. J Rheumatol.

[10] Ohta Y, Tsuchihashi T, Kiyohara K, Oniki H (2013). Increased uric acid promotes decline of the renal function in hypertensive patients: a 10-year observational study. Intern Med.

[11] Zhu P, Lin F, Lin C, Hong D, Lin K, Chen H, et al. Effect of hyperuricemia on the blood pressure response to antihypertensive agents in hospitalized elderly patients. J Cardiovasc Med (Hagerstown). 2012;13(11):741–6. 10.2459/JCM.0b013e328358527c 22964647

[12] Flack JM, Duncan K, Ohmit SE, Quah R, Liu X, Ramappa P (2007). Influence of albuminuria and glomerular filtration rate on blood pressure response to antihypertensive drug therapy. Vasc Health Risk Manag.

[13] Mazzali M, Hughes J, Kim YG, Jefferson JA, Kang DH, Gordon KL (2001). Elevated uric acid increases blood pressure in the rat by a novel crystal-independent mechanism. Hypertension.

[14] Turgut F, Kasapoglu B, Kanbay M (2009). Uric acid and long-term outcomes in CKD. Am J Kidney Dis.

[15] Bellomo G, Venanzi S, Verdura C, Saronio P, Esposito A, Timio M, et al. Association of uric acid with change in kidney function in healthy normotensive individuals. Am J Kidney Dis. 2010;56(2):264–72. 10.1053/j.ajkd.2010.01.019 20385436

[16] Ficociello LH, Rosolowsky ET, Niewczas MA, Maselli NJ, Weinberg JM, Aschengrau A (2010). High-normal serum uric acid increases risk of early progressive renal function loss in type 1 diabetes: results of a 6-year follow-up. Diabetes Care.

[17] Dawson J, Jeemon P, Hetherington L, Judd C, Hastie C, Schulz C (2013). Serum uric acid level, longitudinal blood pressure, renal function, and long-term mortality in treated hypertensive patients. Hypertension.

[18] Shan Y, Zhang Q, Liu Z, Hu X, Liu D (2010). Prevalence and risk factors associated with chronic kidney disease in adults over 40 years: a population study from Central China. Nephrology (Carlton).

[19] Malaguarnera M, Vacante M, Russo C, Dipasquale G, Gargante MP, Motta M, et al. A single dose of rasburicase in elderly patients with hyperuricaemia reduces serum uric acid levels and improves renal function. Expert Opin Pharmacother. 2009;10(5):737–42. 10.1517/14656560902781972 19351224

[20] Goicoechea M, de Vinuesa SG, Verdalles U, Ruiz-Caro C, Ampuero J, Rincón A (2010). Effect of allopurinol in chronic kidney disease progression and cardiovascular risk. Clin J Am Soc Nephrol.

[21] Kanbay M, Ozkara A, Selcoki Y, Isik B, Turgut F, Bavbek N (2007). Effect of treatment of hyperuricemia with allopurinol on blood pressure, creatinine clearence, and proteinuria in patients with normal renal functions. Int Urol Nephrol.

[22] Han M, Park HC, Kim H, Jo HA, Huh H, Jang JY (2014). Hyperuricemia and deterioration of renal function in autosomal dominant polycystic kidney disease. BMC Nephrol.

[23] Ležaić V, Zlatić N, Zogović J, Žarković B, Živković B, Žakula D (2011). Prevalence of renal insufficiency in individuals with obesity. Srp Arh Celok Lek.

